# The effect of ostensive communication on immediate and delayed memory of novel and familiar action patterns

**DOI:** 10.3758/s13421-025-01799-6

**Published:** 2025-11-03

**Authors:** Cristina I. Galusca, Liuba Papeo, Luca L. Bonatti

**Affiliations:** 1 Center for Brain and Cognition, Universitat Pompeu Fabra, Ramon Trias Fargas, 25-27, 08005 Barcelona, Spain; 2 LIP/PC2S, Université Grenoble Alpes, 38000 Grenoble, France; 3 Institut des Sciences Cognitives Marc Jeannerod - UMR 5229, Centre National de la Recherche Scientifique (CNRS) and Université Claude Bernard Lyon1, Villeurbanne, France; 4 ICREA, Pg. Lluís Companys 23, 08010 Barcelona, Spain

**Keywords:** Eye contact, Ostension, Action imitation, Functional action, Novel action

## Abstract

Actions are often learnt incidentally by observing other individuals. How aspects inherent to the social context in which an action is seen affect action learning remains poorly understood. Here we study the effect of a special social signal, the eye gaze of the demonstrator, on the immediate and delayed memory and execution of observed actions. In Experiment 1, healthy young adult volunteers (*N* = 37) watched short videos of a demonstrator performing novel actions with novel objects only once, and were tested for immediate and delayed recall of those actions through action execution. Overall, novel actions on novel objects were recalled more accurately if, during demonstration, the demonstrator had repeatedly gazed towards the participant (direct gaze) versus towards an object (control condition). Experiment 2 (*N* = 41) investigated the immediate and delayed recall of novel and familiar object-use actions performed on familiar objects. In this condition, which involved familiar information, direct gaze had no effect or was even detrimental for recall. These findings show that direct gaze benefits incidental learning and retention of novel information only (novel actions paired with novel objects in Experiment 1), and interferes with the retention of familiar information (familiar objects paired with familiar actions in Experiment 2). This study suggests a role of ostensive eye contact in incidentally learning new actions. At the same time, it limits its beneficial role to a specific type of learning scenario, one involving only novel (as opposed to familiar) information, unveiling the selective function of ostensive eye contact in the transmission of novel cultural behaviors.

**Supplementary Information:**

The online version contains supplementary material available at 10.3758/s13421-025-01799-6.

## Introduction

Throughout their lifetime, humans learn a large repertoire of actions stipulating how to interact with their environment, and with other members of their culture. Acquiring this repertoire is not a straightforward task. On the one hand, learning arbitrary conventions (e.g., *waving* to greet someone, or *toasting* at social gatherings) poses a challenge because the form of the actions is not predictive of their meaning, the object upon which they are performed, or the context in which they usually appear. On the other hand, even for actions performed to achieve an instrumental goal (e.g., drinking water), only a subset is performed on objects with clear affordances (e.g., a glass); the physical properties of many objects do not cue their intended function (e.g., chopsticks). To complicate matters further, multiple actions can be associated with the same object (e.g., one can *drink* or *toast* with a glass) and multiple objects can be associated with the same action (e.g., one can *eat* with *chopsticks* or with *a fork*). Observing others performing actions in social contexts plays a crucial role in learning the repertoire of actions for appropriate use within one’s own culture. However, little is known about how the social context in which an action is seen affects learning new actions; particularly, how different social signals (e.g., eye contact, prosody, or ostensive gestures) contribute to successfully learning actions, and whether those signals have different effects depending on the type of actions and objects involved.

The theory of natural pedagogy has emphasized the contribution of social signals (or *ostensive-communicative signals*) to the fast learning of cultural information (Csibra & Gergely, 2006). People spontaneously display ostensive signals (e.g., eye contact, gaze, or pointing) to highlight culturally relevant aspects that are important for a novice to acquire. In turn, learners are more likely to interpret demonstrations co-occurring with (vs. without) ostensive signals as learning episodes that introduce relevant information, generalizable to future events (Csibra & Gergely, 2006, 2009, 2011; Csibra, 2010). Multiple studies have demonstrated the role of ostensive signals in the acquisition of category-relevant object properties (Marno, Davelaar, & Csibra, 2014, 2016; Yoon, Johnson, & Csibra, 2008), and words (Axelsson, Churchley, & Horst, 2012). In the action domain, it has been shown that imitation is more accurate in the presence of ostensive signals, in infants (Brugger, Lariviere, Mumme, & Bushnell, 2007; Csibra & Gergely, 2009; Hernik & Csibra, 2015; Király, Csibra, & Gergely, 2013), and adults (Wang et al., 2011a, 2011b).

Here we focus our investigation on one signal, eye contact (or direct gaze), which unambiguously conveys the intention to communicate and whom the communicative message is addressed to. We selected this signal for two reasons. First, from birth, humans are sensitive to this social signal, and show a visual preference for individuals who engage in eye contact (Farroni, Csibra, Simion, & Johnson, 2002). Second, direct gaze is ubiquitous in natural social interactions throughout the life span and across cultures. In one-to-one social interactions, people spend about a third of the time engaging in eye contact (Argyle & Ingham, 1972). Given the early response to eye contact, its pervasiveness in human interactions, and the role of ostensive signals in the quick transmission of culturally relevant information, here we address the potential role of eye contact in learning a particular type of cultural behavior, object-directed actions (Csibra & Gergely, 2009). While we know that eye contact modulates different aspects of action imitation and understanding, none of the previous studies addressed how this signal affects the memory for actions.

The link between ostension and long-term memory has been explored by a rich literature on joint attention development, and its fundamental role in social learning has been highlighted. Joint attention, the coordinated attention of two individuals towards a third object or an event, is in itself an important ostensive cue. Multiple developmental studies have shown that from the first months of life, events attended jointly by teachers and novices are better memorized by novices than events attended by novices on their own. For instance, 9-month-old infants looked longer at toys when the experimenter alternated their gaze between the infant and the toy, compared to toys gazed at by an experimenter who never made eye contact with the child (Striano et al., 2006). Kopp and Lindenberger (2011) familiarized infants with objects that varied in the degree to which they were jointly attended by an experimenter. Using event-related potentials, they demonstrated that at 9 months, the frequency of joint attention events was correlated with the amplitude of the positive slow wave (PSW, linked to memory processes in infancy), which may indicate enhanced long-term memory for jointly attended novel objects. In another event-related potentials study, social cues were shown to impact how 18- to 21-month-old toddlers learnt novel words (Hirotani et al., 2009). Infants who were taught new words in a joint attention condition showed more integration difficulties when taught words were paired with incongruent words. This line of research mostly focused on the connection between joint attention and word learning. Effects on memory induced by joint attention have also been reported in preschoolers. Haden and colleagues (2001) studied how the type of mother-child interactions during social events affected 3-year-olds’ memory for those activities immediately and after a 3-week delay. At both time intervals, children remembered better aspects of activities that had been jointly attended to by the dyad, compared to those attended by either the mother or the child.

So far the link between joint attention and memory has mainly been studied in the domain of word learning or verbal recollection of events. But humans also learn a vast amount of actions throughout their lifetime and most of this learning happens through observing others perform them. Yet, to date, research on the role of joint attention in action acquisition has been scant; notably for the purpose of the current study, it is even unclear whether joint attention has an overall effect independent of domain and circumstances, or whether its effect is modulated by the type of actions and objects involved. Here, we started exploring these questions, investigating how eye contact influences the retention of actions performed on objects, and whether its role is ephemerally apparent only in immediate recall, or whether it has a more stable influence on memory, also influencing delayed recall. We manipulated: (1) object and action familiarity, i.e., the existence of previous knowledge about objects or actions involved in the acquisition scenario; and (2) the presence versus absence of a social signal, such as the demonstrator’s eye contact. Familiarity is known to play a fundamental role in the processing of many kinds of information, including actions. Even the brain networks involved in action imitation do not indicate the presence of one single mechanism; indeed, imitation of familiar actions recruits an indirect semantic route, because familiar actions have been previously learnt and are stored in long-term memory. However, novel actions can only involve a direct route linking the visual input to the motor output, and they circumvent long-term memory storage (Rumiati & Tessari, 2002; Tessari & Rumiati, 2004). Because the representation of familiar and novel actions may be separate in the brain, it is reasonable to assume that ostension impacts action processing differentially depending on its familiarity. The current experiments were conceived to explore this hypothesis. We generated videos in which an actress performed a set of object-use actions. Eye contact was manipulated between participants: in the Eye Contact condition, the actress recurrently shifted her gaze from the camera (direct gaze toward the observer) to the object; in the No Eye Contact condition the actress recurrently shifted her gaze between the table (averted gaze) and the object, but never looked directly to the camera (i.e., toward the observer). Experiment 1 studied how ostension affects memory for novel action-object pairs, mimicking a typical scenario in the process of cultural acquisition – the incidental learning of novel actions associated with novel objects. Experiment 2 studied how novel and familiar actions were memorized when paired with familiar objects, examining the role that eye contact plays in: (a) acquiring and recalling the new usage of a familiar object (i.e. learning a novel action performed with a familiar object already associated with another action in memory), and (b) recalling the usage of familiar objects, where no novel information was presented. The theory of natural pedagogy (Csibra & Gergely, 2009) suggests that eye contact would benefit the retention of novel actions, particularly in typical learning scenarios, which pair novel actions with novel objects. Additionally, we explored the effect of eye contact in recalling familiar information (i.e., familiar actions paired with familiar objects) or partially familiar information (i.e., novel actions paired with familiar objects). Since ostension may have a role in cultural acquisition, and thus on memory, we also explored how stable any measurable effects are in memory.

Finally, to address possible differences between the memory for actions and other types of incidental information, both experiments included a control event. Because ostension facilitates the encoding identity-relevant novel object features (i.e., shape), but does not generalize to other types of content such as object extrinsic properties (i.e., location; Marno, Davelaar & Csibra, 2014), our control consisted in hiding the object in one of two possible locations, a kind of information for which ostension should not affect retention.

## Experiment 1

We evaluated the effect of eye contact on immediate and delayed memory for two types of consecutive events. The first event, and of main interest, was a novel action consisting of a sequence of hand and arm movements, involving a novel object. In the second event, shown right after the first event, the object was placed in one of two boxes in front of the demonstrator (the hiding location). The latter event was included to address the specificity of the effect of eye contact on the memory for actions as opposed to any type of information (i.e., memory for a location).

### Methods

#### Participants

Thirty-seven adults (M_age_ = 23.62 years; SD = 4.9 years; 54% women) with no known auditory or visual impairments or psychological or neurological problems, participated in the experiment. In the absence of previous studies with a similar design to ours, Experiment 1 was exploratory with respect to the sample size and its results were used for power calculations for Experiment 2.

Participants gave their informed consent prior to participation, in accordance with the guidelines of the Declaration of Helsinki, and were compensated at the end of the experiment at a rate of 8 € per hour.

#### Stimuli and apparatus

We created a set of videos of an actress (the demonstrator) performing novel actions on novel objects.

**Figure 1.**
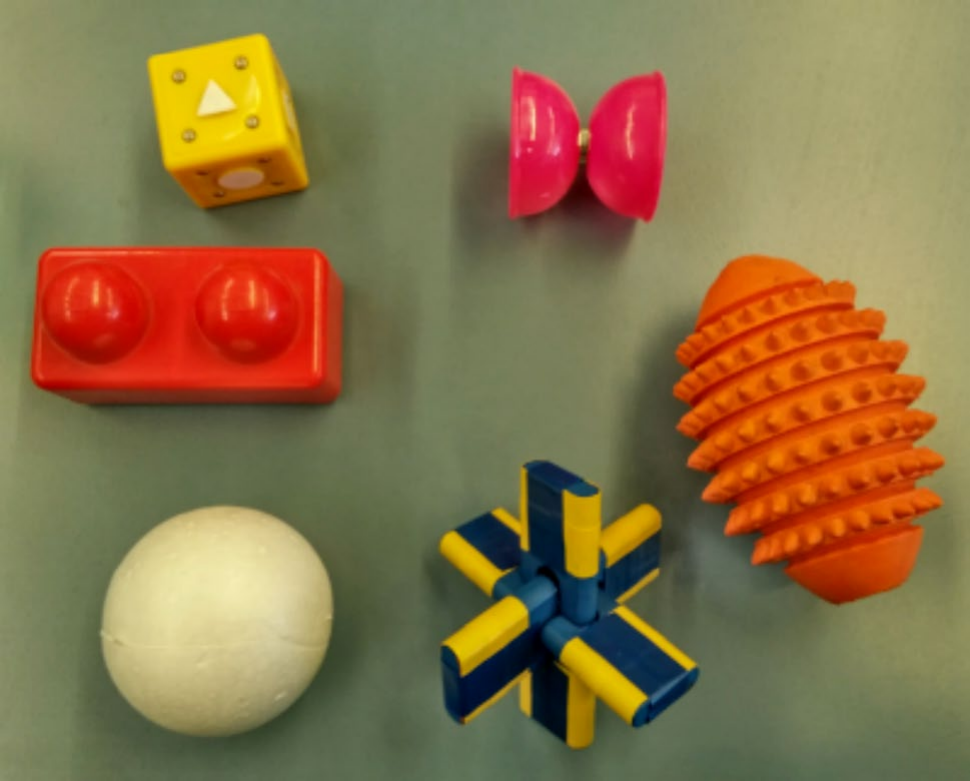
Novel objects used in Experiment 1

*Novel objects* were all comparable in size, but had different colors and shapes, to be easily distinguished from one another (Fig. 1). They had no known or obvious function, to avoid any potential association with familiar object-directed actions.

*Novel actions* were sequences of hand and arm movements with no obvious meaning. Movements were selected from a database of meaningful and meaningless gestures evaluated

by a large sample of participants (Agostini, Papeo, Galusca, & Lingnau, 2019).

**Fig. 2:**
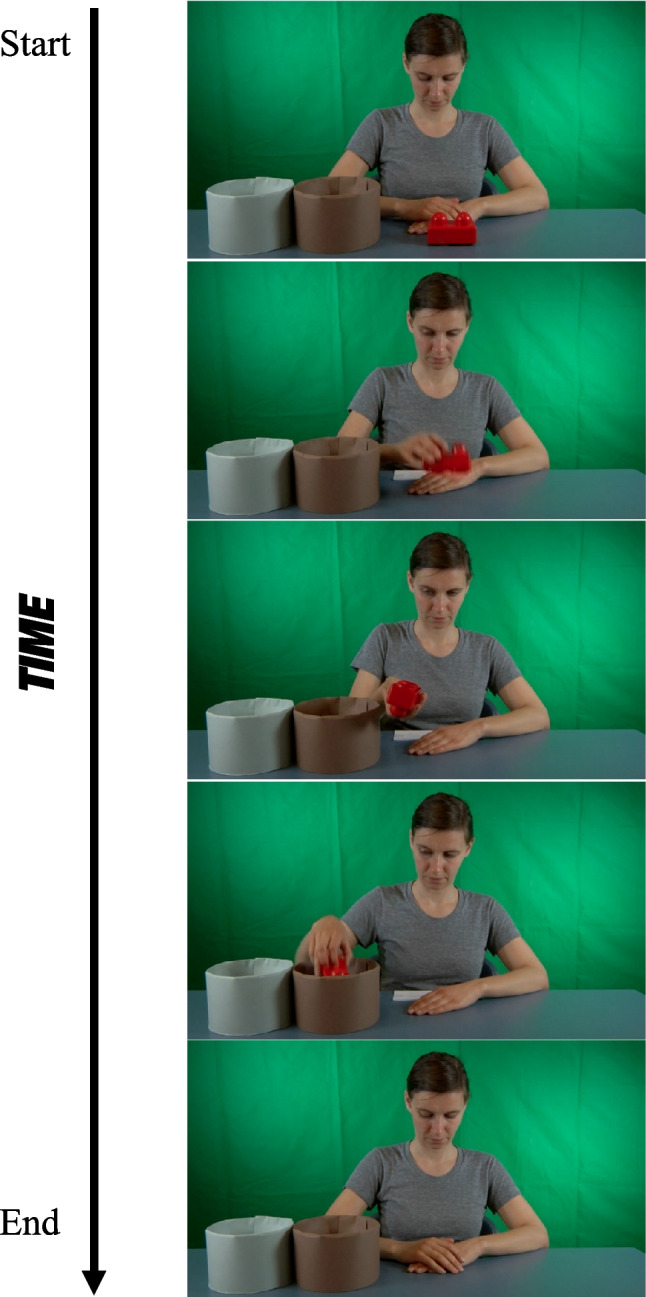
Example screenshots from the No Eye Contact condition (Experiment 1) presenting the sequence of events in each video

*Videos* presented the actress from the waist up, against a green background, sitting at a table. In all the videos, the actress wore the same clothes and displayed a neutral facial expression. Each video started with a novel object in the middle of the table and two boxes on the right side of the actress, who was in a resting position, with her hands crossed on the table. Two sets of videos were recorded corresponding to the two experimental conditions: Eye Contact (EC+) and No Eye Contact (EC-). For the EC+ condition, each video presented the following sequence of events: (1) the actress looked straight into the camera, as if making eye contact with the observer; (2) she grabbed the object and showed it to the camera, while shifting gaze between the object and the camera twice; (3) she performed the novel action, during which she shifted gaze between the object and the camera two to three times; (4) she placed the object in one of the boxes; and finally, (5) she returned to the initial resting position. In the EC- condition, everything was identical except that, instead of looking into the camera, the actress looked down towards a paper on the table, as if she was reading instructions. She never looked at the camera but she performed the actions gazing toward the object or the table (see Fig. 2 and videos S1–S4 the Online Supplementary Material).

For each object and condition (EC+ and EC-), four videos were recorded, to counterbalance the association between the object, the action, and the box. For example, in the video O1A1B1, action A1 was performed with O1, and the object was placed in box B1; for the same object O1, three more videos were recorded: O1A1B2 (same action A1 with same object O1, this time placed in box B2), O1A2B1 (a new action A2 with same object O1 placed in box B1), O1A2B2 (action A2 with same object O1 placed in box B2; videos S1–S4, Online Supplementary Material). The four videos were assigned to four different lists, each seen by different participants. A total number of 48 videos were included (24 for the EC+, and 24 for the EC- conditions), and divided in eight lists of six items each (either all EC+ or all EC-). Videos were recorded with a Panasonic AG-HPX172 camera with a 1,080-pixel resolution; they were edited to remove the sound, and matched in length (mean = 191.93 seconds, range 171–223 frames at 25 fps), with QuickTime 7 Pro.

The experiment was run on an iMac and controlled with Psyscope X B77 (https://cbclab-online.upf.edu/rico/psyscope/). The iMac was connected to a JVC LT-46DZ7 46-in. LCD screen where videos were shown, in full screen (visual angle of 70°), one at a time. The large screen allowed for the presentation of the actress approximately real-life size, about 1 m away from the participant.

#### Procedure

Participants were tested in a sound-attenuated room. The experiment included two sessions. The first session had two phases: Presentation and Immediate Test Phase. During the Presentation, participants sat on a chair, in front of a table with a mouse. The chair was 1 m away from the experimental screen, and height-adjusted to align the participant’s eyes to the demonstrator’s eyes. Before the experiment, participants were only instructed to carefully watch the videos. They were not informed what to pay attention to in the videos and they were not explicitly instructed to memorize the actions for delayed recall and performance. To continue to the next video, participants had to click the mouse. Participants watched six videos, once each, presented in random order across participants.

Each participant was randomly assigned to either the EC+ or EC- conditions and to one of the four lists of the respective condition. At the end of the Presentation, participants were tested on three out of the six objects shown during the Presentation. The remaining three objects, not included in the Immediate Test Phase of the first session, were used in the Test Phase of the second session. Thus, each object was tested only once, either in the first or the second session, to avoid test effects on learning, previously shown to enhance memory performance even in the absence of feedback (Carpenter, Pashler, Wixted, & Vul, 2008). We counterbalanced the order in which the two object-lists were tested across participants. At the beginning of the Immediate Test Phase, the experimenter entered the room and placed the two boxes on the table, together with three of the objects shown in the videos. The screen was switched off and the mouse was removed from the table. Participants were instructed to reproduce the actions on the three objects, as shown in the videos. The test was recorded for offline analysis using a webcam placed on the experimental screen (for an example, see video S5, Online Supplementary Material). Participants had no time limit to reproduce the action.

The second session took place exactly 7 days later in the same room. This interval was chosen based on memory literature showing that retention reduces logarithmically, with the steepest decay in the 7 days following the first presentation. After a week, memory decays at a much slower rate. Furthermore, 1 week is the shortest interval to capture long-term retention (Cepeda, Vul, Rohrer, Wixted, & Pashler, 2008; Loftus, 1985). The second session started directly with the Test Phase, without demonstration. Participants were recorded while performing the actions with the three objects, as they remembered them.

#### Rating

Participants’ performance was evaluated offline by a rater unaware of the hypotheses and purposes of the study. A second independent rater evaluated 25% of the actions performed by participants. Inter-observer agreement was excellent and reached consensus in 93% of the cases (Cohen’s k = 0.86). Rating criteria were based on Rumiati and Tessari’s (2002) criteria for the evaluation of action production performance in a standard neuropsychological test. That is, for each object, the action produced by the participant was assigned to one of the following cases (see Table S6 in the Online Supplementary Material for more details):

- *Recognizable Action-Object Pair:* The positions of the hand and arm and the movements produced by the participant with a given object were similar to those shown in the video with that same object.
- *Substitution:* For a given test-object, the participant produced one of the actions seen during the Presentation Phase, but not the one associated with that object.
- *Unrecognizable:* The movement produced for a given object matched neither the associated action nor any other action seen during the Presentation Phase.
- *Omission*: No movement was performed for a given object.

As for the hiding location, if the participant placed a given object in the same box as shown in the Presentation Phase, the response was given a score of 1 (correct); otherwise, it was assigned a score of 0 (incorrect).

### Results

We focused our analyses on the following three measures. First, we computed the Proportion of Recognizable Action-Object Pairs, quantifying how many action-object pairs were correctly retained. Second, we computed the Proportion of Recognizable Actions, that is, the sum of Recognizable Action-Object Pairs and Substitutions. The former measure evaluated the memory for action-object pairs; the latter evaluated the memory for novel actions, irrespective of whether the action was retained in memory with the correct object. Finally, we quantified the Proportion of Correct Hiding Locations, as a measure of the memory for an incidental property of novel objects.

For each of these dependent variables, we computed Generalized Linear Mixed Models (GLMM; Baayen, 2008; Bates, Mächler, Bolker & Walker, 2015) with binomial error structure and logit link function to compare adults’ performance between conditions, with the R package *lme4* (Bates, Mächler, Bolker & Walker, 2015). We subsequently used the *emmeans* package to estimate marginal means (Lenth, 2025).

We compared a null model to models containing the variable of primary interest for us, Condition (EC+, EC-); Condition and Session (Immediate, Delayed); Condition, Session, and participant Gender; and Condition, Session, and their interactions as fixed effects, with the binary factors (Condition, Session, and Gender) effect-coded using sum contrasts prior to fitting. Participants were always included as a random effect. For model selection, we compared the models with likelihood ratio tests, and selected the model that revealed effects and had the lower Aikake information criterion (Cohen et al., 2013).

**Fig. 3:**
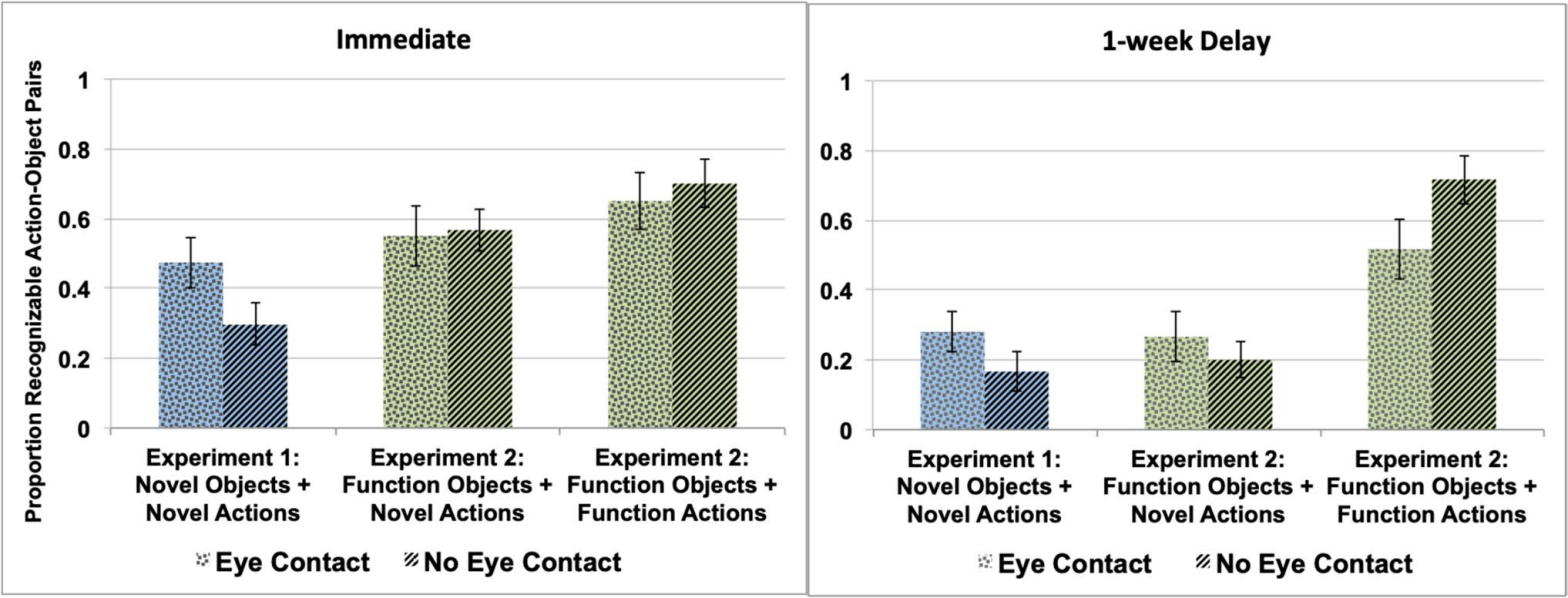
The Proportion of Recognizable Action-Object Pairs for the Novel Objects in Experiment 1, and for the Functional Objects in Experiment 2 split by Condition (EC+/EC-), for the Immediate and 1-week Delay Sessions. The error bars represent one standard error from the mean

*Recognizable Action-Object Pairs.* The model including participant gender as a fixed effect did not add any explanatory power (χ2 = 0.20, Df = 1, *p* = 0.656), hence gender did not enter the selected model. The model comprising the main effects of Session and Condition was the best at explaining variation in the data for this dependent variable. The model yielded significant main effects of Condition (ß = 0.39, SE = 0.19; z = 2.08; *p* = 0.038) and Session (ß = −0.43, SE = 0.16; z = −2.70; *p* = 0.007). Participants remembered a higher number of action-object pairs in the EC+ condition (M_EC+_ =.377, SE =.046) than in the EC- condition (M_EC-_ =.231, SE =.041). The main effect of Session reflected a memory decay from the Immediate (M_Immediate_ =.387, SE =.046) to the 1-week Delay Test (M_1-week Delay_ =.225, SE =.040; see Fig. 3 and Table 1). The fact that there was no gain in adding the interaction in the model indicates that the advantage of the EC+ group carried over the two sessions.

**Table 1:** The Proportion of participant responses corresponding to each rating category (recognizable action-object pairs, substitutions, unrecognizable actions and omissions) for each experiment, condition and session

**Experiment 1**: Novel Objects + Novel Actions		Session 1		Session 2	
		EC+	EC-	EC+	EC-
	Recognizable Action-Object Pairs	0.48	0.30	0.28	0.17
	Substitution	0.39	0.33	0.35	0.26
	Unrecognizable	0.43	0.44	0.60	0.57
	Omission	0.09	0.26	0.12	0.26
**Experiment 2**: Familiar Objects + Novel Actions		Session 1		Session 2	
		EC+	EC-	EC+	EC-
	Recognizable Action-Object Pairs	0.55	0.53	0.27	0.20
	Substitution	0.12	0.22	0.17	0.22
	Unrecognizable	0.17	0.25	0.27	0.32
	Omission	0.28	0.17	0.47	0.48
**Experiment 2**: Familiar Objects + Familiar Actions		Session 1		Session 2	
		EC+	EC-	EC+	EC-
	Recognizable Action-Object Pairs	0.48	0.70	0.52	0.72
	Substitution	0.05	0.05	0.00	0.03
	Unrecognizable	0.10	0.10	0.02	0.07
	Omission	0.27	0.20	0.47	0.22

**Fig. 4:**
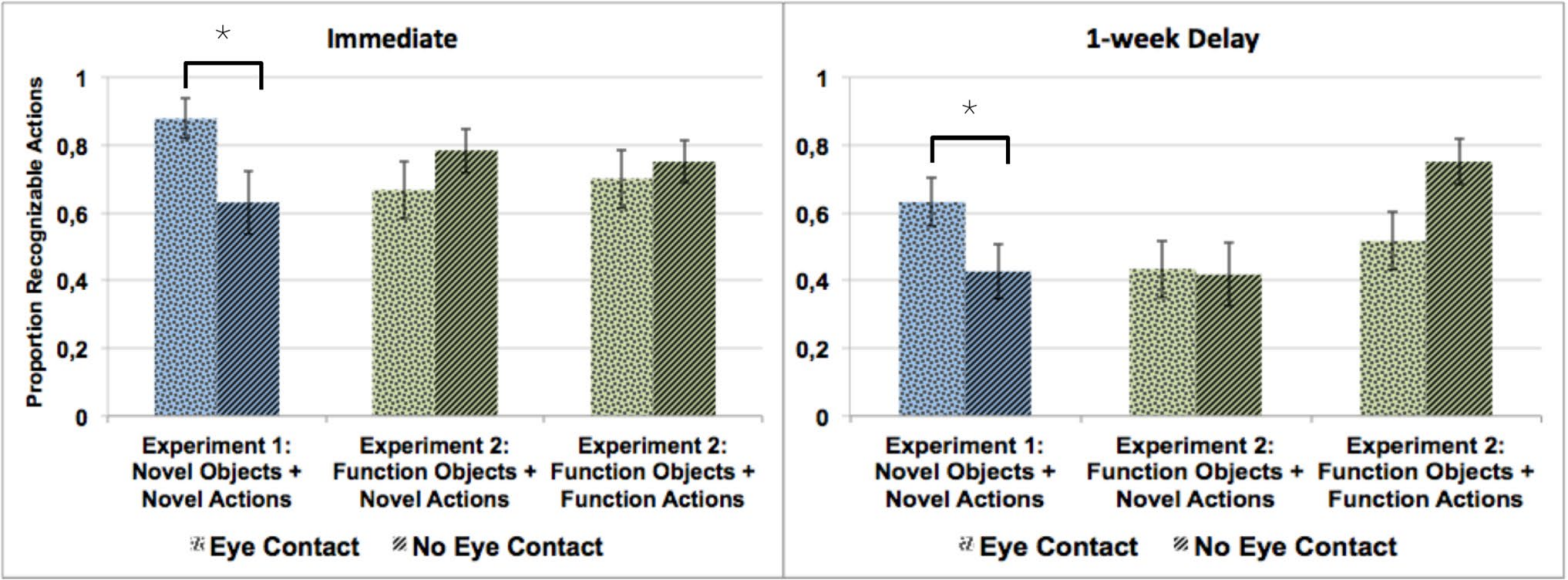
The Proportion of Recognizable Actions for the Novel Objects (Experiment 1) and Functional Objects (Experiment 2) split by Condition (EC+/EC-) for the Immediate and 1-week Delay Sessions. The error bars represent one standard error from the mean

*Recognizable Actions.* Again, adding participant gender as a factor did not account for variation in the data (χ2 = 0.85, Df = 1, *p* = 0.355) and was not included in the selected model. The best model comprised the main effects of Session and Condition as fixed effects. The model yielded significant main effects of Condition (ß = 0.77, SE = 0.33; z = 2.32; *p* = 0.020) and of Session (ß = −0.78, SE = 0.20; z = −3.98; *p* < 0.001). On average, more actions were remembered in the EC+ condition (M_EC+_ =.754, SE =.041) than in the EC- condition (M_EC-_ =.528, SE =.048). The main effect of Session revealed a better performance in the Immediate Test (M_Immediate_ =.757, SE =.041) than in the 1-week Delay Test (M_1-week Delay_ =.532, SE =.048; see Fig. 4 and Table 1). Again, the fact that these variables did not interact indicates that the advantage of the EC+ group applied to both the immediate and delayed recall.

*Hiding Location.* No model was better at explaining variation in the data than the null model. Indeed, performance was at chance in both Conditions and Sessions (one-sample *t-*tests: First Session: M_EC+_ =.596, *p* =.260; M_EC-_ =.556, *p* =.476; Second Session: M_EC+_ =.561, *p* =.434; M_EC-_ =.463, *p* =.586).

### Discussion

In Experiment 1, we associated a novel object with a novel action and a hiding location. The relationship between the object and the information associated with it was arbitrary in both cases: the properties of the objects (e.g., color, shape, or texture) were not indicative of any action to be performed upon them or a hiding location. We evaluated the role of ostensive communication (i.e., direct gaze) in the immediate and delayed memory for the actions, the action-object pairs and the hiding locations, presented to participants only once. We report two main findings.

First, we found an advantage in learning novel actions for novel objects in an ostensive-communicative scenario. The memory for novel actions was better if the actions had been demonstrated with direct gaze. This result is remarkable, considering that participants had one single exposure to each novel action. These findings are compatible with the theory of natural pedagogy, which argues that ostensive signals indicate relevant novel information, and highlight opportunities for learning (Csibra & Gergely, 2009; Csibra, 2010), eliciting an expectation of relevance in the receivers (Sperber & Wilson, 1986). Even when the meaning or causal structure of a novel action is cognitively opaque, as in the current scenario, ostension can guide the interpretation of an action as culturally relevant and potentially indicative of an object’s function or customary manner of handling it (Gergely & Csibra, 2006). These circumstances, reproduced in our experiment, may mimic the conditions in which individuals learn arbitrary conventional behaviors of their culture.

Second, we found no advantage in retaining the location information in the Eye Contact versus No-Eye Contact condition. While we do not know why the hiding location escaped the beneficial effect eye contact, this finding rules out that the effects on memory for actions and action-object pairs reflected an overall increase of attention induced by direct gaze. Thus, these results may suggest a specific role of eye contact in learning novel objects and their use, but it may also suggest that learning the location was too difficult in the current task. 

We note that the performance for the hiding location was at chance, and propose two possible explanations: (1) as suggested, the task was simply too difficult for participants, or (2) in a learning scenario where novel objects and actions were presented, the hiding location was much less salient and participants prioritized the learning of the novel action-object pairing. The current experiment does not allow us to disambiguate between these interpretations. However, Experiment 2 introduces familiar information and could partially confirm one of these alternatives.

## Experiment 2

Experiment 1 investigated how novel actions are retained when performed on novel objects, depending on the social context. In Experiment 2, we considered the case of familiar objects, which already have a known usage (i.e., they are associated with a familiar action) but here were shown in a new usage (i.e., they become associated with a novel action). We investigated how direct gaze affected the memory for associations between familiar objects and actions representing their natural use versus novel arbitrary actions. Novel and familiar actions are suggested to be processed by different mechanisms and brain networks: in action imitation, familiar actions are processed via a semantic route, where the motor output is guided by the activation of an action representation stored in memory, corresponding to the observed action to imitate; novel pantomimes are processed by a slower, direct route that directly transforms the visual input into motor output, without the mediation of semantic representation (Rumiati & Tessari, 2002, Tessari & Rumiati, 2004). Thus, familiar and novel actions are processed differently and ostension may selectively highlight information to be learnt.

### Methods

#### Participants

Forty-one adults (M_age_ = 24.83; SD = 4.5 years; 66% females) participated in this experiment. Forty participants were included in the final analysis; one participant was excluded for not participating in the second session. Given the similarity in designs, we used the structure and the coefficients of Experiment 1, obtained during the analysis of Recognizable Action-Object Pairs, to estimate the power of Experiment 2 by running permutations (200 permutations per each number of participants), using the R packages *designR* (Rabe, Kliegl, & Schad, 2023) and *simr* (Green & MacLeod, 2016). The estimated power for Experiment 2, for about 40 participants, is about 75%.

#### Stimuli, apparatus, and procedure

The videos used in this experiment were designed and edited in the same way as in Experiment 1. Instead of novel objects, here we used six familiar objects with distinct and familiar functions (hereafter, *functional objects:* brush, key, eraser, stapler, sponge, and car). The functional objects were similar in size, but differed in color and shape. Two actions were presented consecutively for each functional object, within the same video: a novel and a functional action. The functional action was a demonstration of the object’s canonical use (e.g., for the key, rotating the key rightward, as if turning it into a lock to open a door). The novel action was selected from a set of meaningless action sequences (Agostini, Papeo, Galusca, & Lingnau, 2019). Novel and familiar actions were presented consecutively for each object. We opted for this design for several reasons. First, we presented the novel and familiar actions together for the same object to avoid ceiling effects in the retention of functional actions. Also, since our main aim was to test participants’ selective retention of different types of information from ostensive and non-ostensive demonstrations, our design provided a direct comparison. Second, novel actions were always presented before familiar ones to minimise the effect of prior knowledge (the familiar action on the familiar object) in the encoding of the novel information (novel action on familiar object). The hidden location was counterbalanced across objects. Apparatus and procedure were otherwise identical to Experiment 1.

#### Rating

Videos were evaluated offline for the novel action and the functional action following the same criteria as in Experiment 1 (see Table S6, Online Supplementary Material, for details). A second rater scored 25% of the participants actions and reached a 93% agreement for the novel actions (Cohen’s kappa = 0.86) and 95% agreement for the familiar actions (Cohen’s kappa = 0.9), with the main rater. As in Experiment 1, we assigned a binary score to the correct/incorrect choice of the hiding location.

### Results

As in Experiment 1, we analyzed the three dependent variables of interest separately: Proportion of Recognizable Action-Object Pairs, Proportion of Recognizable Actions, and Proportion of Correct Hiding Locations. For each dependent variable, we compared models containing Condition (EC+, EC-), Session (Immediate, Delayed), and/or ActionType (novel, function) as fixed effects (coded using sum contrast); models containing their two-way interactions; and models containing their three-way interaction. We always included Participants as a random factor. Criteria for model selection were identical to those in Experiment 1.

*Recognizable Action-Object Pairs.* A model including participant gender as a fixed effect did not add any explanatory power (χ2 = 2.70, Df = 1, *p* = 0.100), and hence gender was not included in the final analysis. The best model was the one including Session, Condition, Action Type, and their two-way interactions. This model yielded a main effect of Action Type (ß = 0.65, SE = 0.11; z = 5.9; *p* < 0.001) and Session (ß = −0.51, SE = 0.11; z = −4.6; *p* < 0.001). Participants recalled functional actions better than novel actions (M_Function_ =.646, SE =.031; M_Novel_ =.396, SE =.032; see Fig. 3 and Table 1). As expected, they also performed better immediately than at a 1-week delay (M_Immediate_ =.617, SE =.031; M_1-week Delay_ =.425, SE =.032).

There was also an interaction between Session and Action Type (ß =.35, SE = 0.11; z = 3.21; *p* = 0.001). Post hoc comparisons using a Holm correction for multiple comparisons revealed a decay for novel actions between the immediate and delayed sessions (ß = −1.71, SE = 0.32; z = −5.41; *p* < 0.001). However, there was no decay for functional actions (ß = -.32, SE = 0.30; z = −1.06; *p* = 0.585).

*Recognizable Actions.* The model comprising session, condition, action type, and the three-way interaction between them was the most explanatory model. As for the previous analyses, in a separate model we also checked participant gender as a fixed effect. This variable did not explain the variation in the data better than the above model (χ2 = 4.91, Df = 2, *p* = 0.767), and we did not include gender in the selected model. The reported model yielded a main effect of Session, as expected (ß = −0.62, SE = 0.12; z = −5.14; *p* < 0.001), due to memory degradation (M_Immediate_ =.73, SE =.029; M_1-week Delay_ =.52, SE =.032). The model also yielded a main effect of Action Type (ß =.32, SE = 0.12; z = 2.76; *p* = 0.006), with a higher number of functional actions recalled compared to novel actions (M_Functions_ =.679, SE =.030; M_Novel_ =.567, SE =.032; see Fig. 4). In addition, the model revealed a significant Session by Action Type interaction (ß =.33, SE = 0.12; z = 2.79; *p* = 0.005). Post hoc comparisons Holm- corrected revealed a decay for novel actions from the immediate to the delayed session (ß = −1.89, SE = 0.34; z = −5.59; *p* < 0.001), but no decay for function actions (ß = −0.58, SE = 0.33; z = −1.74; *p* = 0.24). Interestingly, there was no difference in performance between functional and novel actions in the immediate session (ß = −0.007, SE = 0.34; z = −0.02; *p* = 0.983), but functional actions were significantly better remembered in the delayed session compared to novel actions (ß = 1.30, SE = 0.32; z = 4.04; *p* = 0.002).

Finally, the model revealed a three-way interaction Session × Condition × Action Type (ß = −0.24, SE = 0.12; z = −2.05; *p* = 0.040). Post hoc Holm-corrected comparisons revealed that in the EC- condition the decay from the immediate to the delayed session was smaller for function actions than for novel actions (ß = 2.27, SE = 0.68; z = 3.33; *p* < 0.001). However, in the EC+ condition no such difference arose; the decay from the first to the second session was equivalent for the two types of actions (ß = 0.35, SE = 0.64; z = 0.54; *p* = 0.590; Fig. 4).

*Homogeneity of Functional Actions.* One of the main variables manipulated in this experiment was Action Type (functional vs. novel). In the case of the selected familiar functional actions, we assumed participants knew these canonical functions. One possibility is that some actions within this category were less recognizable or familiar to our participants and reduced the effect for the whole category. To test this possibility, we performed a Bartlett’s test to explore the homogeneity of variance in accuracy rates for Recognizable Functional Action-Object Pairs among the six objects. The results revealed no difference in variance between different objects (*K*^*2*^ = 2.83, *df* = 5, *p =*.725), suggesting that participants treated functional actions in a similar way.

*Hiding Location.* As in Experiment 1, after seeing actions performed on objects, participants saw in which of two boxes objects were hidden. Action Type had no relevance for this dependent variable and was not included in the analysis. We analyzed participants’ responses for object hiding locations (one response per object). The model that best explained the data comprised Session, Condition, and their interaction. A separate model including participant gender as a fixed effect added no information and hence gender was dropped from the selected analysis. The selected model yielded a strong trend towards an effect of Session (ß = −0.31, SE = 0.16; z = −1.9; *p* = 0.056), with better recall of the hiding location in Session 1 (M_Immediate_ =.73, SE =.04; M_1-week Delay_ =.63, SE =.03). However, there was an interaction between Session and Condition (ß = −0.44, SE = 0.16 z = −2.75; *p* = 0.006). Post hoc Holm-corrected comparisons revealed that in the EC+ condition there was a decay from the first to the second session (ß = −1.50, SE = 0.48; z = −3.13; *p* = 0.011), but in the EC- condition there was no such decay (ß = 0.28, SE = 0.43; z = 0.64; *p* = 1).

Performance was above chance level in the First Session for both conditions (M_EC+_ =.833, *p* <.001, *Cohen’s d* = 1.21; M_EC-_ =.633, *p* =.049, *Cohen’s d* =.47), while in the Second Session this was the case only for the non-ostensive demonstration (M_EC+_ =.5833, *p* =.235, *Cohen’s d* =.28; M_EC-_ =.683, *p* =.037, *Cohen’s d* =.5).

### Discussion

The first result of Experiment 2 was the presence of an overall advantage in remembering and producing functional actions compared to novel actions. The second result was that memory for novel actions decayed from the First to the Second Session, but this was not the case for the memory for functional actions. Overall, there were also fewer substitutions for functional compared to novel actions (see Table 1). These results suggest that the recall of the functional actions used in this study is highly stable in memory, suffering almost no interference from the co-presentation of other (novel) actions. Thirdly, unlike Experiment 1, in which novel actions paired with novel objects were better retained when demonstrated with direct gaze, in Experiment 2 eye gaze had no effect on remembering novel actions performed on familiar objects, and it even reduced the memory for functional actions. These results circumscribe the role of eye contact to learning scenarios involving *novel* action-related information. Because ostensive social interactions generate an expectation to learn (Csibra & Gergely, 2009; Csibra, 2010), one possible explanation could point at an infelicitous role of eye contact when associated with already learnt information: employing ostensive signals such as eye contact while demonstrating familiar information (i.e., familiar object-use actions), such as the function of well-known objects, may violate pragmatic expectations of relevance, and this may have interfered with the encoding or recall of those functional actions. This interpretation paves the way to further research exploring how the learning circumstances modulate the role and benefits of ostension.

Finally, Experiment 2, unlike Experiment 1, revealed an effect of eye contact on the memory for the hiding location. Immediately after the presentation, memory for the hiding location was particularly accurate in the EC+ (vs. EC-) condition. Here we used familiar objects, which may suggest that when kind-relevant information, function, use, and label are already established in memory, ostension benefits learning and retention of extrinsic features, such as location. Our findings for the hiding location are rather inconsistent between the two experiments, and our interpretations of these effects are post hoc. Future research should systematically study the encoding of location as a function of object familiarity and the novelty of the information presented.

## General discussion

The current research investigated the effect of nonverbal ostensive communication, expressed through eye contact, on incidental learning and remembering of different types of action sequences (novel vs. functional-familiar) performed on different types of objects (novel vs. functional-familiar). In particular, we inspected how immediate and delayed memory is affected by the demonstrator’s gaze pattern, while watching novel or functional actions paired with novel or familiar objects. Communicative signals, in general, are paramount for the acquisition of cultural information (Csibra & Gergely, 2009). Here, we focused our study on eye contact, a major nonverbal cue used by a speaker to convey the intention to communicate to a specific addressee (Csibra & Gergely, 2009, 2011; Senju & Johnson, 2009), and spontaneously interpreted as a signal to learn novel information in adults, children, and infants (Király, Csibra, & Gergely, 2013; Marno, Davelaar, & Csibra, 2014, 2016; Yoon, Johnson, & Csibra, 2008). Our findings encourage a number of considerations on how processing and learning motor behaviors are affected by gaze direction, interacting with action and object familiarity.

### The role of ostension

We found that ostension has a different effect on memory depending on the type of material to learn and retain. Memory for novel actions paired with novel objects is enhanced when the demonstration involves direct gaze. Instead, when novel actions are paired with objects that already have a known associated function, direct gaze has no effect. Moreover, memory for the familiar functional actions performed over the typical functional objects is negatively impacted by ostension. It is important to note that our paradigm captures incidental learning, in the absence of explicit instruction to remember the observed actions, more akin to how cultural behaviors are acquired. Understanding the relevance of the observed behavior and remembering it relies heavily on the observer’s capacity to interpret ostensive communication.

These findings suggest that eye contact *selectively* benefits learning when acquiring completely novel information (i.e., novel actions with novel objects), but not when the information to remember is partially (novel actions performed over familiar-functional objects) or entirely known (i.e., familiar functional actions performed over the canonical objects). This suggests that, when a demonstrator ostensively indicates the importance of encoding novel information, novel actions produced on novel objects are more likely to be encoded and remembered. In this sense, ostension has a crucial role in highlighting the importance of completely novel information, the relevance of which is unknown to participants. When novel actions were paired with familiar objects, we found no effect of ostension. One plausible interpretation is that adults, just like children, may adopt a “design stance,” and expect objects to be designed for a single purpose: their canonical function. Since they already knew the primary functions of familiar objects, adults already possessed the most important information about that object kind (Greif et al., 2006; Kelemen & Carey, 2007), and were less susceptible to learning novel information, even when that information was presented ostensively. Another possibility is that, more generally, ostension benefits the memory for more difficult types of information (novel actions paired with novel objects), rather than easier types of information (novel actions paired with familiar objects). While the current interpretations remain speculative, future research should address whether ostension has a different effect in learning actions in children, compared to the pattern we observed in adults. Children have more limited repertoires of actions compared to those of adults, and actions and objects are less rigidly associated in children’s memory (i.e., a phenomenon known as functional fixedness by which older children and adults are unable to see novel uses for familiar objects; German & Defeyter, 2000). Thus, ostension may interact differently with object and action novelty in children.

Additionally, we suggest that, in the third context, where functional actions were paired with familiar objects, ostension is unnecessary, if not irrational, as it signals the importance of learning something that adult observers already know (e.g., how to use a key). As such, the ostensive act violates the presumption of relevance in the addressee (Sperber & Wilson, 1986). In this perspective, our findings suggest that violations of presumption of relevance may even be detrimental to learning and retention. However, the current study does not have a baseline condition to establish whether our result is a detrimental effect of eye contact, or instead a beneficial effect of no eye contact. Follow-up research should further investigate the nature of this effect. We stress that our findings do not imply that eye contact erased the memory for familiar object functions, or that it caused a permanent interference with the semantic memory of those items; rather, we suggest that eye contact may have interfered with the encoding of functional actions in episodic memory. While a wealth of studies (including the current Experiment 1) have demonstrated the benefit of ostension for learning, this is among the first of these studies to highlight the limits of ostension, showing when it can interfere with learning and memory. Future studies fully crossing over action novelty, object novelty, and ostension will be particularly useful to corroborate the current pattern of results. An alternative interpretation of the lower recall of functional actions presented ostensively is that participants may have looked longer at the demonstrator’s face, when the information presented was already known, especially in the eye-contact condition. An interesting line for future research would be to measure the relative attention to a demonstrator’s face and to the object, as a function of action and object familiarity. This interpretation is supported by De Felice, Vigliocco, & Hamilton (2021), who recently reported similar effects: in a pre-recorded learning situation, adults remembered novel facts better when the teacher’s face was not seen, only their hands were seen. However, they found the opposite effect during live online teaching, suggesting that ostension improves learning during live interactions, but it has the opposite effect for observational learning (De Felice et al., 2023).

We finally point out that the differential effects of ostension depending on the information to be learned and recalled help to rule out an interpretation of eye contact as a mere signal that boosts attention or the saliency of the stimuli (Gredebäck, Astor, & Fawcett, 2018; Szufnarowska, Rohlfing, Fawcett, & Gredebäck, 2014). The attention modulation account predicts that the presence of attention-grabbing cues (here, eye contact) increases attentional levels toward the present information, irrespective of context or the information content. Unspecific attentional enhancement would lead to improved memory performance for all the material that had received attention, regardless of familiarity. This model cannot account for the lack of effect of direct gaze on memory for novel actions performed on familiar objects, or for the detrimental effect of direct gaze on memory for familiar functional actions performed on familiar objects.

Importantly, in our study one demonstrator showed all the actions on novel and familiar objects. While our findings reveal that social interactions benefit the learning of novel behaviors, we also know that adults, and also children, do not learn indiscriminately from all individuals; they are selective in whom they trust and who they learn from. Previous research documented an early developing tendency to seek out information from knowledgeable or reliable individuals (Corriveau & Harris, 2009; Koenig & Harris, 2005). Even 3-year-old children trust individuals who provide accurate information over those who provide inaccurate or contradictory answers (Koenig & Harris, 2005). Thus, future research should complement our current findings and address how demonstrator social identity (e.g., gender, age), and their perceived competence influence the effect ostensive signals have in learning new cultural behaviors.

### The interplay between action familiarity, object familiarity, and mode of presentation

We found that direct gaze promotes the reproduction of novel actions paired with novel objects, only after a single exposure to the pairs and even after a temporal delay. At the same time, direct gaze was negatively associated with the processing of functional actions for known objects. This result is relevant to our understanding of how the direct and semantic routes of action imitation may work. These routes recruit shared neural circuitry (Decety, Chaminade, Grezes, & Meltzoff, 2002; Iacobini, Wood, Brass, Bekkering, Mazziotta & Rizzolatti, 1999), but also distinct neural mechanisms (Decety et al., 1997; Grezes, Costes, & Decety, 1998). Our behavioral results may license some tentative speculations about the interplay of these mechanisms. The results are consistent with the possibility that novel actions activate the dorsal stream, crucial for visuomotor transformations. By contrast, familiar actions, which involve semantic processing, may particularly activate the ventral stream, crucial for object recognition. The dorsal stream, responsible for processing novel actions, overlaps with brain structures involved in gaze processing in attentional and social tasks, such as the posterior superior temporal and fronto-parietal regions (Allison, Puce & McCarthy, 2000). Anatomical proximity or overlap might favor the interplay between visuomotor transformation mechanisms implemented in the parieto-frontal dorsal stream and gaze processing in the fronto-parietal network. This interplay might underlie the advantage of direct eye contact in processing of novel action-object pairs. In contrast, familiar actions presented during eye contact may elicit simultaneous activation of the ventral and dorsal streams, yielding interference between the two processes. While speculative, this hypothesis opens yet another avenue for integrative research.

In summary, the effect of ostensive communication on recalling motion sequences, even after very brief presentations, highlights the role of social signals in action processing and learning. Our main finding is that ostensive signals affect the retention of object-use actions differently, depending on action and/or object familiarity. Selective effects of ostensive communication may apply to information outside the action domain, reflecting a specific function of social signals in the cultural transmission of novel information.

## Supplementary Information

Below is the link to the electronic supplementary material.

Supplementary file1 (DOCX 10 KB)

## Data Availability

The datasets generated and/or analyzed during the current study are available on the Open Science Framework (https://osf.io/szfvj/).

## Code availability

The action annotation scheme used to rate participants’ action productions is available in the Supplementary Material. The code used to analye the current study is available on the Open Science Framework (https://osf.io/szfvj/).
