# Supplementary material for: The effect of ostensive communication on immediate and delayed memory of novel and familiar action patterns
Source: Mem Cognit. 2025 Nov 3;54(4):1116–29. doi: 10.3758/s13421-025-01799-6 (PMC13253588; doi:10.3758/s13421-025-01799-6)
Supplement: Supplementary file 1 — Supplementary file1 (DOCX 10 KB) [file 13421_2025_1799_MOESM1_ESM.docx]

**Supplementary Material**

Supplementary videos are available at:

<https://osf.io/t8vkw/?view_only=8151a4bc767c4d00b7994f82bc7aefd4>:

**S1** - Video used in Experiment 1 in the EC- condition for the novel object O1, novel action A1 and box B1

**S2** - Video used in Experiment 1 in the EC- condition for the novel object O1, novel action A1 and box B2

**S3** - Video used in Experiment 1 in the EC+ condition for the novel object O1, novel action A1 and box B1

**S4** - Video used in Experiment 1 in the EC+ condition for the novel object O1, novel action A1 and box B2

**S5** - Video example of a participant’s performance in Experiment 1, Session 1

**S6** - Table describing the criteria for rating the novel and functional actions in Experiments 1 and 2.

**S7** - Video used in Experiment 2 in the EC- condition for familiar object O1

**S8** - Video used in Experiment 2 in the EC+ condition for familiar object O1

Table S6

| **Imitation Type** | **Action Error** | **Action Error Description** |
| --- | --- | --- |
| **Recognizable** | None | the performed action is very similar to the observed action |
|  | Orientation | the overall movement is recognizable but it is performed in the wrong direction (e.g. left to right instead of right to left; rotation performed in the wrong direction) |
|  | Perseveration | it involves the extra repetition of an action or part of an action previously presented |
|  | Reduction | it involves the reduction of a repetitive cycle of the same action to a shorter output action or to one single cycle |
|  | Planar | the overall movement is recognizable but the height of the object is altered: the action is performed in the air instead of on the table or simply higher/lower than in the original video |
|  | Amplitude | the overall movement is recognizable but the size of the movement is altered to either larger or smaller than original |
|  | Distance from body | the overall movement is recognizable but the action is performed in a different position with regard to the participant's body (e.g. it is either closer or farther away ) |
|  | Side of body | the overall movement is recognizable but the action is performed on the wrong side of the body, e.g. on the left side instead of on the right side |
|  | Position of arm/hand | the overall movement is recognizable but the posture of the hand or arm is modified |
| **Substitution** | Novel action with novel action | replacing the meaningless action corresponding to the object with a meaningless action corresponding to another object in the list |
|  | Functional action with functional action | replacing the meaningful action corresponding to the object with a meaningful action corresponding to another object in the list |
| **Unrecognizable** | Unrecognizable novel action | the response involved a movement that the raters failed to recognize as part of the action repertoire in the experiment |
| **Omission** | Omission | no action was performed |
